# Synergistic taurine and methionine supplementation enhances growth and cholesterol regulation in *Totoaba macdonaldi*

**DOI:** 10.1007/s10695-025-01590-z

**Published:** 2025-11-07

**Authors:** Omar Aguillón-Hernández, María Teresa Viana, José A. Mata-Sotres, Ceres A. Molina-Cárdenas, Fernando Barreto-Curiel

**Affiliations:** 1 Facultad de Ciencias Marinas, Universidad Autónoma de Baja California (UABC), Carr. Tijuana-Ensenada 3917 Fraccionamiento Playitas, 22860 Ensenada, Baja California, México; 2 Instituto de Investigaciones Oceanológicas, Universidad Autónoma de Baja California (UABC), Km 107 Carretera Tijuana-Ensenada Fraccionamiento Playitas, 22860 Ensenada, Baja California, México; 3 Departamento El Hombre y Su Ambiente, Universidad Autónoma Metropolitana, Unidad Xochimilco, Coyoacan, Mexico City, Mexico; 4 Centro de Investigación Científica y de Educación Superior de Ensenada (CICESE), Ensenada, Baja California, México

**Keywords:** Taurine, Methionine, Amino acids, Cholesterol, *Totoaba macdonaldi*

## Abstract

Totoaba (*Totoaba macdonaldi*) aquaculture offers economic and ecological advantages. However, its culture still relies on fishmeal in diets because alternative protein sources show reduced productive performance. The current study assessed the impact of low concentrations of methionine and taurine together with alternative proteins, on the productive performance of *T. macdonaldi* over a 60-day experimental period. Four diets were formulated for this purpose, a basal diet (D-BD), the basal diet with methionine (D-MET), the basal diet with taurine (D-TAU), and the basal diet with methionine and taurine (D-MET + TAU). The present experiment used a randomized design. One hundred forty-four juveniles (41.0 ± 0.5 g in weight) were randomly distributed in 12 tanks (500 L) in triplicate groups to assess biological indices, cholesterol content, hepatic gene expression, and the synthesis and transport of taurine. The statistical analysis revealed that the dietary treatments D-MET and D-TAU positively affected the growth rate, whereas their interaction resulted in a significantly higher growth (*p* < 0.05). The expression of the *igf-1* gene in the liver increased and showed a positive interaction. When TAU and MET were limited, there was an observed overexpression of *csad* in hepatic tissue. Diets supplemented with TAU showed a decrease in total cholesterol level, whereas cholesterol level in the liver increased with MET supplemented alone. Total TAU content in fish tissues was significantly higher when both TAU and MET were supplemented. In conclusion, *T. macdonaldi* exhibits a limited capacity, for TAU synthesis, and MET limitation appears to restrict growth potential.

## Introduction

Aquaculture production in Mexico, similar to other regions worldwide, aims to reduce the overfishing of economically valuable fish species while protecting native species. A particular focus is on *Totoaba macdonaldi,* an endemic fish species from Northwestern Mexico that has been overfished due to illegal fishing practices. Despite the challenges, totoaba overfishing has incentivized research projects that enhance the understanding of its reproduction and cultivation, both economic and ecological benefits for the region. Totoaba is a carnivorous fish with promising culture potential due to its fully controlled reproduction, and its rapid growth rate, with low disease occurrence. Furthermore, Furthermore, the high nutritional quality of its meat generates strong market demand (Fuentes-Quesada et al. 2023).

Nevertheless, the culture of carnivorous fish species presents several challenges, particularly the commercial feed formulations that meet their nutritional requirements to achieve commercial-size production. Many studies have reported that replacing fishmeal with plant protein sources, in particular, soybean meal as an alternative protein can lead to enteritis and reduced performance (Fuentes-Quesada et al. 2023; 2018), and even green liver syndrome in other carnivore fish species (Takagi et al. 2006a).

Generally, replacing fish meal for plant sources raises various concerns (Urán et al., 2008; Lin et al., 2017; Jannathulla et al. 2019; Liu et al., 2021; Makhdoom et al. 2024), making animal protein sources from rendering process a more favorable alternative. Nonetheless, the rendering process often reduces the concentration of crucial amino acids such as methionine (MET) and taurine (TAU). MET is an essential amino acid that contains sulfur and serves as one of the building blocks for protein synthesis, while TAU, an amino sulfonic acid, can be found in free form. MET is present in lower amounts within animal renderer sources, such as poultry by-product meal and TAU is sometimes absent (Fuertes et al. 2013; Hekmatpour et al., 2018; Siddik et al. 2019). Consequently, both amino acids must be supplemented in the diet.

The metabolic functions of both amino acids are different. When MET is present at lower levels than required, protein synthesis halts through the General Control Non-depressible 2 (GCN2) mechanism (Wang et al. 2016b). Beyond serving as a building block for protein synthesis, MET also acts as a precursor of S-adenosyl MET, cysteine, and TAU in many organisms (Oda, 2006).

TAU, derived from sulfur-containing amino acids (Burini et al. 2018) is found in high concentrations in most animal tissues (Voss et al. 2004). It plays critical roles in several essential biological processes, functioning as a neurotransmitter and osmolyte. In marine species, TAU is involved in the homeostasis between corporal fluids and the marine environment, as well as the bile salt synthesis. Bile salts are a crucial emulsifier for lipid digestion in the intestine (Hayes and Sturman 1981). Although fish require high levels of TAU, it is not involved in protein synthesis (Park et al. 2002; Lunger et al. 2007; Johnson et al. 2015). Both TAU and cholesterol are precursors of sodium taurocholate, the primary molecule in bile acid (Yun et al. 2012). Diets formulated with fishmeal typically contain 300 to 700 mg/kg of cholesterol, whereas diets high in plant protein sources range from 50 to 110 mg/kg (Norambuena et al., 2013). Generally, the effects of reducing cholesterol by plant-based diets appear to be more detrimental than those in diets based on animal protein sources or semi purified diets (Norambuena et al., 2013). Observations indicate that one of the most significant functions of TAU relates to cholesterol metabolism, which directly impacts bile acid formation. Nevertheless, various studies in fish have demonstrated that TAU also influences cellular function, regulating oxidative stress and immune activity (Aguillón-Hernández et al. 2017; Gunathilaka et al. 2019; Liu et al. 2022).

The maintenance of TAU levels is regulated by the cysteine sulfonic acid decarboxylase (CSAD) system and TAU transporter (Taut), with the liver serving as the primary organ for de novo TAU synthesis (Chang et al. 2013). Additionally, the liver is responsible for the elimination of TAU via bile (Yun et al. 2012). Recent studies have demonstrated that dietary supplementation of both taurine and methionine can significantly enhance productive performance in various fish species. For example, Hoseini et al. (2022) reported that the combined addition of taurine and methionine to plant-based diets improved blood parameters, immune responses, and growth in Persian sturgeon (*Acipenser persicus*). Similarly, in rainbow trout (*Oncorhynchus mykiss*), a diet containing 7.5 g/kg of taurine and 2.5 g/kg of methionine improved productive performance, immunological response, and hepatic antioxidant capacity (Aguillón et al., 2020). Peter et al. (2024) showed that the inclusion of 15 g/kg of taurine and 10 g/kg of methionine in the diet of pangasius (*Pangasianodon hypophthalmus*) enhanced protein retention, improved body composition, and reduced plasma glucose and triglyceride levels; however, excessive methionine supplementation decreased growth. In European sea bass (*Dicentrarchus labrax*), 12-week feeding trials using diets low or high in methionine with or without 1% taurine supplementation did not affect growth, feed utilization, or body composition, but high methionine levels increased hepatic catalase (CAT) and glutathione peroxidase (GPx) activity, as well as total and reduced glutathione in the gut, suggesting modulatory effects on antioxidant status (Coutinho et al. 2017). These studies indicate that the combined supplementation of methionine and taurine tends to produce greater benefits than supplementing either one alone, particularly in diets high in plant proteins, and can additionally improve key metabolic aspects such as lipid metabolism and antioxidant capacity. Therefore, the objective of the present study is to evaluate the relevance of dietary TAU and MET on growth and cholesterol content in *Totoaba macdonaldi.*

## Materials and methods

### Diet formulation and preparation

Four isoproteic and isolipidic diets were formulated with varying levels of MET and TAU in a randomized design, containing 50% crude protein (CP) and 10% crude fat (CF). The basal diet (D-BD) was not supplemented with MET and TAU but complied with the recommended content for marine fish (National Research Council, 2011). The other three diets included one with MET at 1% (D-MET), a third with TAU at 1% (D-TAU), and the fourth with MET and TAU at 1% each (D-MET + TAU).

Nutritional requirements for *Totoaba macdonaldi* have been reported for several key nutrients, including protein, lipids, lysine, and taurine (González-Osorio et al. 2012; Rueda-López et al. 2011; Madrid et al. 2019; Satriyo et al. 2017). For taurine, the established requirement comes from studies using washed fishmeal, which helped keep the background levels of other amino acids similar to those used in protein requirement experiments (Satriyo et al. 2017). However, there is evidence that using plant proteins may increase the taurine requirement, since certain fibers can bind bile salts and alter their metabolism (Xie et al. 2025; Cai et al. 2020; Ni et al. 2021; Liland et al. 2013).

As for methionine, there are no specific requirement studies for totoaba, nor has its potential role as a taurine precursor been determined in this species. Given these gaps, the approach in this experiment was to supplement taurine and methionine at levels comparable to those naturally present in fishmeal, as outlined in National Research Council (2011) recommendations. These values served as a practical starting point for diets largely containing plant-derived ingredients. No preliminary trials were conducted beforehand; thus, the National Research Council (NRC) guidelines were used as the best available reference). Additionally, lysine was supplemented at 0.5% in all treatments to maintain a similar level to that found in fish meal (National Research Council, 2011).

### Amino acid composition

The amino acid composition (AAs) was determined using defatted samples (by Soxhlet extrac-tion, according to AOAC 1995) that were homogenized and ground. Briefly, 400 μL of HCl 6 N containing 0.06% phenol was added to 10 mg of the pulverized samples. The mixture was then digested in a closed vial under a nitrogen atmosphere for 18 h at 113 °C to produce free AAs. The digested samples were diluted at 25 mL with deionized water, and 1 mL of 2.5 mM α-aminobutyric acid (AABA) was added as an internal standard. The samples were filtered through a Teflon filter of 0.45 μm and stored in a nitrogen atmosphere at -30 °C. All the hydro-lyzed samples were derivatized using an AccQ Kit tag reagent (Cohen et al., 1994), injected in a Waters high-performance liquid chromatography (HPLC) system, utilizing a reverse phase col-umn C-18 (3.9 × 150 mm; Waters™, USA). The extract was eluted using a water-acetonitrile gradient at constant temperature (37.5 °C). The derivatized amino acids were measured with a fluorescence detector (series Waters 474, Milford, USA.) with excitation at 250 nm and emission at 395 nm, using a standard AA solution from 18.5 to 300 pmol; Waters Corporation prod No. WAT088122). Standard curves were obtained for each amino acid. Tables 1 and 2 show the amino acid profile and the proximal diet composition.

**Table 1:** Dietary formulation given as g/Kg, and proximate composition of experimental diets fed to *Totoaba macdonaldi* juveniles. Diets were supplemented with methionine, taurine and both compared to a basal diet

	Treatments			
*Ingredients*	D-BD	D-MET	D-TAU	D-MET + TAU
Poultry by-products meal^a^	237.0	232.0	232.0	229.0
Sardine fish meal^b^	96.0	94.0	94.0	93.0
Gelatin^c^	63.0	61.0	61.0	61.0
Soybean concentrate^d^	248.0	243.0	243.0	240.0
Wheat gluten	50.0	49.0	49.0	48.0
Sardine fish oil^e^	60.0	60.0	60.0	60.0
methionine^f^	0.0	10.0	0.0	10.0
Taurine^f^	0.0	0.0	10.0	10.0
Corn starch	211.0	215.0	215.0	213.0
Rovimix^g^	25.0	25.0	25.0	25.0
Stay C^g^	2.0	2.0	2.0	2.0
Lysine^h^	5.0	5.0	5.0	5.0
Sodium benzoate	2.0	2.0	2.0	2.0
BHT	0.1	0.1	0.1	0.1
***Proximate composition in percentage***				
Crude protein	51.2	49.9	51.4	49.4
Crude fat	10.6	10.2	10.2	9.7
Ash	7.7	7.7	7.7	7.6

^a^ National Renderers Association, USA. ^b^ Proteínas marinas y agropecuarias S.A. de C.V. Guadalajara, Jalisco, Mexico. ^c^ Órganos y semillas San Miguel, Ensenada, Baja California, Mexico. ^d^ 63% CP; HP300, Nutrivance nutrition advance Midwest-Ag Enterprises, Inc. Minnesota, USA. ^e^ 68% CP; Monterrey sardine, Mexico. ^f^ Future foods materias primas, Tlalnepantla, Estado de México, Mexico. ^g^ DSM nutritional products México S.A. de C.V., Mexico. ^h^ ADM, Mexico

**Table 2:** Amino acid profile from experimental diets fed to *Totoaba macdonaldi* juveniles. Diets were supplemented with methionine, taurine and both compared to a Basal diet

Treatments				
*Amino acids*	D-BD	D-MET	D-TAU	D-MET + TAU
ASP	4.27	4.39	4.71	4.22
SER	2.36	2.52	2.57	2.32
GLU	8.85	8.58	9.06	8.06
GLY	4.58	4.35	4.48	4.29
HIS	0.84	0.97	0.86	0.9
ARG	3.3	3.92	3.34	3.26
THR	1.93	1.81	1.71	1.77
ALA	2.94	2.77	2.81	2.75
PRO	4.43	4.1	4.4	4.11
VAL	2.54	2.39	2.59	2.36
**MET**	**0.8**	**1.51**	**0.65**	**1.97**
**TAU**	**0.26**	**0.17**	**1.05**	**1.05**
LYS	4.07	3.79	3.8	3.78
ILE	2.37	2.24	2.34	2.11
LEU	4.03	3.95	4.1	3.69
PHE	2.3	2.29	2.23	2.29

### Experimental design

A total of 144 *Totoaba macdonaldi* juveniles were donated by CREMES (Centro Reproductor de Especies Marinas del Estado de Sonora, Hermosillo, Sonora, Mexico). The experiment was performed in the facilities of IIO (Instituto de Investigaciones Oceanológicas) at the Universidad Autónoma de Baja California, México. The organisms were acclimated for 15 days and were fed a commercial diet (Otohime C2, Marubeni Nisshin Feed Co., Ltd. Tokyo, Japan).

After the acclimatization, the juveniles were randomly distributed in 12 500-L tanks with sea water (34 PPT), with a stocking density of 12 fish per tank. There were four treatments, each replicated three times. Water was recycled through a biofilter (PolyGeyser®; drop-in-air pneumatic filter Model PG7 International Filter Solutions, TX, USA) coupled to a reservoir. During the feeding assay, the water temperature was maintained at 26 °C ± 1.0 °C using a 6500-watts heater inside a deposit tank equipped with a PT100 sensor. Dissolved oxygen levels were kept above 5 mg/L, nitrite levels below 0.5 mg/L, and ammonia levels were kept at 0–0.25 mg/L. Five percent of the water was replenished daily to maintain the optimal water quality parameters. Each diet was randomly assigned in three tanks, and the organisms were hand-fed until apparent satiety four times a day (08:00, 11:00, 14:00, and 17:00 h) for a duration of 60 days. The average weight of each experimental unit was recorded at the beginning and on day 60 (at the end of the experiment), by dividing the sum of each individual weight by the total number of fish. The general yield indices were then determined according to the following formulas:

$$\begin{array}{c}Gained\;weight\;percentage\:=\:\lbrack(Final-initial\;weight)\;\ast100/Initial\;weight\rbrack\\Specific\;growth\;weight\;(SGR)\:=\:(Final\;weight-Initial\;weight)\;\ast100/t\;(in\;days)\\Protein\;efficiency\;rate\;(PER)\:=\:g\;of\;humid\;corporal\;weight\;gain/g\;of\;ingested\;protein\\Thermal\;growth\;coefficient\;(TGC)\:=\:\lbrack(1/3\;final\;weight\:-\:1/3\;initial\;weight)/(T^\circ\ast Number\;of\;days)\rbrack\;\ast1000\\Condition\;factor\;(CF)\:=\:(Final\;weight/length^3)\;\ast100\\Hepatosomatic\;index\;(HI)\:=\:(liver\;weight/body\;weight)\;\ast100\\Viscerosomatic\;index\;(VI)\:=\:(viscera\;weight/body\;weight)\;\ast100\end{array}$$

At the end of the feeding trial, all organisms were sacrificed following the protocols set by the university’s ethics committee (UABC). The organisms were placed in containers with ice water to reduce their metabolism and induce thermal hypothermia. Subsequently, disconnection was performed through cranial puncture (Canadian Council on Animal Care, 2005). Six fish were collected from each experimental unit for proximal composition analyses and cholesterol content. Additionally, three more fish were sampled for liver tissue to assess gene expression; a small liver sample was stored in RNA-Later.

### Proximal composition

The proximal composition of the whole fish, diets, and tissues was estimated using the methods outlined by the Association of Official Analytical Chemists (1995). Six organisms from each experimental unit were sampled, including whole fish, liver, and muscle tissues, which were individually macerated using a food processor and stored in plastic bags at −30 °C until analyses. The samples were then dried at 60 °C and pulverized with a micro pulverizer (IKA Mills, IKA Works, Inc., NC, USA) before the analysis. Humidity content was determined at a constant dry weight at 60 °C, as indicated before. Total nitrogen content was determined (method 960.52) using the Micro-Kjeldahl method (LABCONCO, Corporation, MO, USA), and crude protein was calculated as %N × 6.25. Total lipid concentration was estimated using the Soxhlet method (method 920.97), and ash content was determined by incineration at 550 °C for six hours (method 942.05). The nitrogen-free extract content was calculated using the following formula:

$$\%\;NFE=\:nitrogen-free\;extract=100-(\%crude\;protein+\%total\;lipid+\%ash)$$

### Cholesterol analysis

Cholesterol content in tissues was determined through solvent extraction (Folch et al. 1957). The non-saponifiable layer of the lipid/fatty acid analysis was silanized by incubating the samples using HTP (hexamethyldisilazane: trimethylchlorosilane: pyridine, 2:1:5, v:v:v) for 60 min in a water bath at 75 °C. A gas chromatography Agilent GC 6850 (Agilent Technologies, CA, USA) equipment was utilize with a dimethylpolysiloxane capillary column (30 m, 0.32 mm in internal diameter and 0.25 μm in field thickness; Agilent Technologies HP-1, CA, USA). The samples were manually injected (1 μL) in splitless mode with the injector and detector temperature set at 280 °C and 330 °C, respectively. The oven temperature program was as follows: 150 °C for 3 min, increase from from 150 °C to 200 °C at a rate of 25 °C/min; then from 200 °C to 280 °C at 5 °C/min; finally, from 280 °C to 295 °C at 1 °C/min. Hydrogen was used as carrier gas at a flow rate of 45 mL/min. Cholesterol concentration in the samples were calculated using pure cholesterol (C8667_Merck, NJ, USA) at varying concentrations to generate a standard curve.

### Taurine content

To determine taurine (TAU) content, whole macerated fish were used individually. Two hundred mg of dry samples were diluted in 1 mL of perchloric acid at 1 M. The samples were homogenized and then centrifuged at 13,000 rpm for 2 min. The supernatant was transferred to a clean test tube and neutralized at a pH 7.0 by adding an equal volume of 2 M KOH solution. A 50 μL aliquot of the solution was taken for analysis.

A commercial kit (Taurine assay kit MET-5071 Cell Biolabs, Inc. San Diego, Ca, USA) containing dioxygenase TAU was used for TAU quantification. The method is based on the enzymatic reaction using α-ketoglutarate as a substrate. The reaction product (aminoacetaldehyde sulfite) was measured at 405 nm in a Multiskan™ 51,119,000 (ThermoFisher Scientific, MA, USA) spectrophotometry, and a standard curve was created with increasing concentrations from 0 to 1000 μmol of TAU to calculate concentration in the samples. The final TAU content was reported as μmol and subsequently transformed to milligrams per gram.

### Gene expression

Individual samples of hepatic tissues from each experimental unit were utilized for gene expression analysis. The individual tissues were preserved in RNAlater (Ambion, Inc.) and processed for total RNA extraction using TRIZOL. The RNA quantity and quality were assessed using electrophoresis and spectrophotometric measurements (Nanodrop® LITE, Thermo Fisher Scientific Inc., Wilmington, USA). Only RNA samples with a OD260nm-OD280nm ratio from 1.90 to 2.10 were used for gene expression quantification.

Total RNA (500 ng) was reversed transcribed in 20 μL reaction using the high-capacity cDNA reverse transcription kit (Applied Biosystems; Carlsbad CA, USA) in a Verity thermocycler with 96-well plates (Applied Biosystems, USA). The reverse transcription program comprised 10 min at 25 °C, 120 min at 37 °C, 5 min at 85 °C followed by maintainance at 4 °C. The qRT-PCR reaction was conducted with 1 ng of cDNA, using sense and antisense primers (200 nM each, indicated in Table 3) and SYBR® Select Master Mix (Applied Biosystems, MA, USA). The 18S gene (GenBank acc. NoHM_754483) served as an internal reference. The control treatment was used as calibrator for all experimental condition and genes studied, including the reference gen and repeated for all analyzed plates. Reactions were performed in 10 μL in MicroAmp Fast Optical 96-well reaction plates (Applied Biosystems) covered with MicroAmp® Optical Adhesive Film (Applied Biosystems). The 2-ΔΔCT method was used to calculate the relative gene expression from liver samples (Livak and Schmittgen 2001) utilizing automated threshold and baseline for determining CT values. Reactions were performed in 10 μL in MicroAmp Fast Optical 96-well reaction plates (Applied Biosystems) covered with MicroAmp® Optical Adhesive Film (Applied Biosystems). The 2^−ΔΔ*C*T^ method was used to calculate the relative gene expression from liver samples (Livak and Schmittgen 2001) utilizing automated threshold and baseline for determining CT values. The PCR conditions were established as follows: an initial denaturation and activation phase at 95 °C for 10 min, followed by 40 cycles of denaturation at 95 °C for 15 s. This was succeeded by annealing and extension at 60 °C for 45 s. Finally, a final fusion stage was conducted, ramping from 60 °C to 95 °C for 20 min to check for the presence of possible primer-dimer artifacts.

**Table 3:** Sequences of the primer pairs used for q-PCR, amplicon size in base pairs (bp), reaction efficiencies (E) and Pearson’s coefficient of determination (R^2^)

Gene (*symbol*)	Fwd sequence (5’—3’)	Rev sequence (5’—3’)	Size (bp)	E	R^2^
*18 s*	CGGTTCTATTTTGTGGGTTTTC	CTTTCGCTTTCGTCCGTCTT	126	0.98	1
*Igf1*	TCCTGTAGCCACACCCTCTC	GGCCATAGCCTGTTGGTTTA	163	0.97	0.99
*taut*	TATCATGCTGCTGCTTCTGG	CACATACATGCCACCTTTCG	187	0.98	0.96
*csad*	TCGCCAAGTACAGCATCAAG	GCAGTCTCCATCAGCACAAA	160	0.95	0.92

For the qPCR optimization, the conditions were set to a primer annealing temperature of 60 °C, with primer and template concentrations at 200 nM and five dilution series (1:10 from 10 to 100 ng RNA input) concentrations. The nucleotide sequence for *18S*, *igf-1*, *csad*, and *taut* nucleotide sequences, were obtained from the GenBank under the accession number HM754483, KR072487, and HQ148721, respectively.

### Statistical analyses

A one-way ANOVA was used to analyze the data, with a significance level set at *p* < 0.05. The analysis was performed using the statistical R software (R Core Team 2021). The source of sulfur compounds was considered the main effect in the model, and the significant differences were identified using a Tukey´s pairwise comparison (*p* < 0.05).

## Results

### Growth performance

The biological index of totoaba is presented in Table 4. Generally, the D**-**MET + TAU treatment exhibited significant increments in final weight (FW), weight gain (WG), thermal growth coefficient (TGC), and specific growth rate (SGR) with values of 138.0 ± 1.3, 233.4 ± 22.6, 1.0 ± 0.1, and 2.2 ± 0.1, respectively. Conversely, the D-BD treatment (control) showed the lowest productive parameters. The feed conversion ratio (FCR) did not show significant differences between treatments (*p*˃0.05). However, the protein efficiency ratio (PER) and the corporal condition factor (CF) exhibited significant differences when comparing the MET and D-MET + TAU treatments to the others (*p* < 0.05). The viscerosomatic (VSI) and hepatosomatic (HSI) indices were not affected by the treatments (Table 4).

**Table 4:** Production performance of juvenile *Totoaba macdonaldi* fed with diets supplemented with methionine, taurine and both compared to a Basal diet. Values represent the average ± standard deviation (*n* = 3); superscripts indicate differences between treatments (*p* < 0.05)

Biological Index	D-BD	D-MET	D-TAU	D-MET + TAU
Initial weight (g)	42.8 ± 1.3	41.6 ± 0.5	41.7 ± 1.1	41.4 ± 1.4
Final weight (g)	91.9 ± 6.8^c^	108.5 ± 7.3^b^	108.6 ± 1.8^b^	138.0 ± 1.3^a^
Weight gain (g)	49.1 ± 6.9^c^	66.9 ± 4.6^b^	66.9 ± 1.3^b^	96.6 ± 8.0^a^
Weight gain (%)	114.9 ± 17.1^c^	160.9 ± 10.1^b^	160.6 ± 3.0^b^	233.4 ± 22.6^a^
FCR	1.3 ± 0.35	1.0 ± 0.0	1.1 ± 0.0	1.0 ± 0.1
TGC	0.6 ± 0.06^c^	0.8 ± 0.0^b^	0.8 ± 0.0^b^	1.0 ± 0.1^a^
SGR (%)	1.4 ± 0.1^c^	1.7 ± 0.1^b^	1.7 ± 0.0^b^	2.2 ± 0.1^a^
PER	1.6 ± 0.3^b^	1.9 ± 0.1^a^	1.7 ± 0.1^b^	2.1 ± 0.0^a^
Cf	1.5 ± 0.1^b^	1.7 ± 0.1^a^	1.6 ± 0.1^b^	1.7 ± 0.0^a^
VSI	3.2 ± 0.3	3.0 ± 0.3	3.2 ± 0.4	3.2 ± 0.2
HSI	1.1 ± 0.2	1.1 ± 0.3	1.0 ± 0.3	1.2 ± 0.1

*TGC*; Thermic growth coefficient; *SGR*; Specific growth rate; *FI*; Feed intake; *PER*; Protein Efficiency Ratio; *Cf*; Condition factor; *VSI*; Viscerosomatic Index

### Proximate composition

Table 5 shows the proximate composition of whole fish, where no significant differences were observed across the treatments. Notably, TAU supplementation positively influenced lipid deposition (*p* < 0.05) with a content of 12.8 ± 1.0 g/100 g with the MET + TAU treatment in the whole fish.

**Table 5:** Proximate composition of whole fish *Totoaba macdonaldi* juveniles fed with diets supplemented with methionine, taurine and both compared to a basal diet. Values represent the average ± standard deviation (*n* = 3); superscripts indicate differences between treatments (*p* < 0.05)

	Treatments				
% Content	Initial	D-BD	D-MET	D-TAU	D-MET + TAU
Crude protein	56.7 ± 1.1	60.5 ± 1.9	61.2 ± 0.8	58.8 ± 2.2	59.9 ± 1.5
Crude Lipids	10.9 ± 0.8	10.2 ± 0.3^b^	10.1 ± 1.2^b^	11.8 ± 1.7^a^	12.8 ± 1.0^a^
Ash	14.3 ± 1.0	14.8 ± 1.8	14.3 ± 0.9	15.1 ± 1.1	14.5 ± 1.1

*CP*, crude protein; *CL*, crude lipids

### Cholesterol and taurine content

Total cholesterol levels in whole fish significantly decreased with the TAU treatment (Table 6), with the lowest concentration was observed in the MET + TAU treatment at 0.02 ± 0.008 mg/g of tissue. TAU resulted in a remarkable 78% reduction in cholesterol level in whole fish. A similar trend was observed in the liver cholesterol content, where the MET treatment indicated a significant increase, while TAU and TAU + MET treatments led to a significant decrease (*p* < 0.05) in total cholesterol (Table 7). The TAU content in whole fish showed significant differences (*p* < 0.05) along treatments, with the highest levels found in the TAU treatment, followed by MET + TAU, MET, and finally by the last D-BD (Table 8).

**Table 6:** Cholesterol content as dry basis in whole body of *Totoaba macdonaldi* juveniles fed with diets supplemented with methionine, taurine and both compared to a basal diet. Values represent the average ± standard deviation (*n* = 3); superscripts indicate differences between treatments (*p* < 0.05)

	D-BD	D-MET	D-TAU	D-MET + TAU
**Cholesterol** (mg/g of tissue)	0.20 ± 0.08^a^	0.34 ± 0.1^a^	0.08 ± 0.01^b^	0.02 ± 0.008^b^
**Cholesterol** (mg/g of fat)	2.2 ± 0.5^a^	3.2 ± 1.1^a^	0.9 ± 0.05^b^	0.2 ± 0.09^b^
**Lipid content** (mg/g of tissue)	100.0 ± 2.8^b^	99.0 ± 7.0^b^	110.0 ± 3.2^a^	123.0 ± 10.0^a^

**Table 7:** Cholesterol content as dry basis in the liver of *Totoaba macdonaldi* juveniles fed with diets supplemented with methionine, taurine and both compared to a basal diet. Values represent the average ± standard deviation (*n* = 3); superscripts indicate differences between treatments (*p* < 0.05)

	D-BD	D-MET	D-TAU	D-MET + TAU
**Cholesterol** (mg/g of tissue)	0.7 ± 0.1^b^	1.2 ± 0.3^a^	0.6 ± 0.2^b^	0.8 ± 0.0^b^
**Cholesterol** (mg/g of fat)	2.7 ± 0.6^a^	3.2 ± 1.2^a^	1.2 ± 0.9^b^	1.4 ± 0.8^b^
**Lipid content** (mg/g of tissue)	275.0 ± 80.0^b^	407.0 ± 100.0^ab^	560.0 ± 94.0^a^	563.0 ± 131.0^a^

**Table 8:** Taurine content as dry basis in whole body of *Totoaba macdonaldi* juveniles fed with diets supplemented with methionine, taurine and both compared to a basal diet. Values represent the average ± standard deviation (n = 3); superscripts indicate differences between treatments (*p* < 0.05)

	D-BD	D-MET	D-TAU	D-MET + TAU
Taurine (mg/g)	1.4 ± 0.6c	4.97 ± 0.4b	16.9 ± 3.8a	13.0 ± 4.2a

### Gene expression

**Fig. 1:**
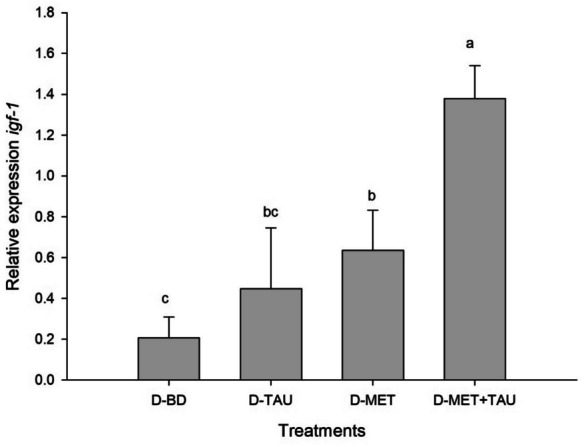
Relative expression of *igf-1* in liver of *Totoaba macdonaldi* juveniles fed with diets supplemented with methionine, taurine and both compared to a basal diet. Values represent the average ± standard deviation (*n* = 3); superscripts indicate differences between treatments in a Tuckey post-hoc test (*p* < 0.05)

**Fig. 2:**
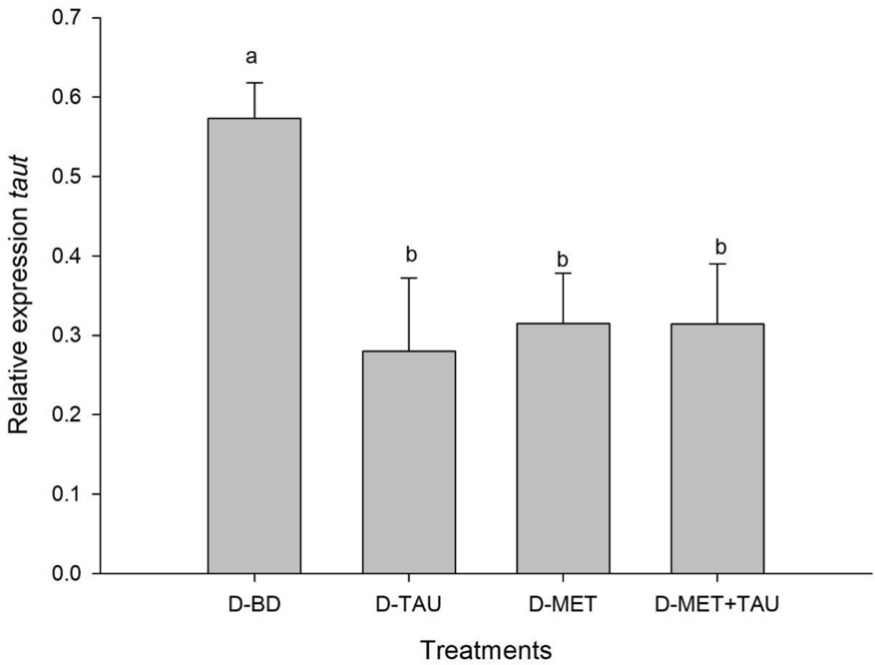
Relative expression of *taut* in liver of *Totoaba macdonaldi* juveniles fed with diets supplemented with methionine, taurine and both compared to a basal diet. Values represent the average ± standard deviation (*n* = 3); superscripts indicate differences between treatments in a Tuckey post-hoc test (*p* < 0.05)

**Fig. 3:**
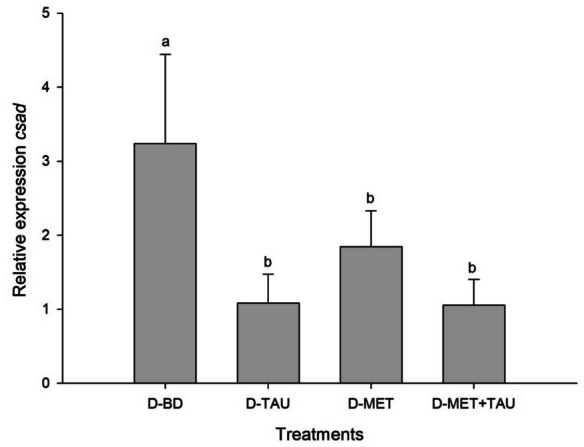
Relative expression of *csad* in liver of *Totoaba macdonaldi* juveniles fed with diets supplemented with methionine, taurine and both compared to a basal diet. Values represent the average ± standard deviation (*n* = 3); superscripts indicate differences between treatments in a Tuckey post-hoc test (*p* < 0.05)

Figure 1 shows the *igf-1* relative expression in the liver. The MET + TAU treatment showed a significant increase compared to the other treatments (*p* < 0.05). The MET treatment also elevated the *igf-1* expression relative to the basal diet (D-BD). Meanwhile, a significant decrease in *taut* expression was observed in all treatments compared to the D-BD in the liver (Fig. 2), which exhibit the highest expression. The *csad* expression in the hepatic tissue decreased with the MET, TAU, and MET + TAU treatments, yielding relative average expression values of 1.2. In contrast, the absence of supplementation led to higher expression, with significant differences compared to the other treatments (*p* < 0.05, Fig. 3).

### Discussion

Dietary methionine (MET) or taurine (TAU) supplementation improved the growth of totoaba, as observed in other species (Candebat et al. 2021, 2023; Li et al. 2021). When provided together, these amino acids produced a synergistic effect, resulting in greater weight gain than with individual supplementation. This agrees with findings in Nile tilapia (*Oreochromis niloticus*) (Urbich et al. 2022) but contrasts with studies in California yellowtail (*Seriola dorsalis*) and European sea bass (*Dicentrarchus labrax*) (Coutinho et al. 2017; García-Organista et al. 2019), where a combined effect was not evident. These interspecific differences suggest that the synergy between MET and TAU is species-dependent.

TAU has been classified as a conditionally essential nutrient in fish due to its multiple physiological functions, including bile salt formation, fat emulsification, osmoregulation, and glucose metabolism (Kim et al. 2008; Takagi et al., 2006c; Zhang et al., 2019). Nevertheless, the present findings indicate that dietary TAU does not decrease MET requirements in totoaba, unlike results reported in rainbow trout (*Oncorhynchus mykiss*) and rice field eel (*Monopterus albus*) (Michelato et al. 2018; Gibson et al. 2007; Hu et al., 2021). This discrepancy may be related to the limited ability of totoaba to synthesize TAU from precursors, a metabolic restriction that has also been described in other marine species (El-Sayed 2014). The endogenous availability of taurine is tightly linked to the expression of genes regulating its biosynthesis and transport, particularly cysteine dioxygenase (*cdo*), cysteic sulfinic acid decarboxylase (*csad*) and the taurine transporter (*taut*), which directly influence physiological status (Martínez-Burguete et al. 2024).

No pathological alterations were observed in any treatment group, suggesting that TAU restriction did not cause liver injury, in contrast to findings by Satriyo et al. (2017) when fishmeal replacement led to histological disorders. Gene expression analysis revealed a significant upregulation of insulin-like growth factor 1 (*igf-1*) in the MET + TAU treatment, consistent with enhanced growth performance. The IGF-1 protein acts as a mediator of growth hormone in tissues such as the liver, promoting protein synthesis and anabolic activity (Bower & Johnston 2010). Its expression in fish is modulated by dietary nutrients, with amino acids and energy sources playing central roles (Bower & Johnston 2010). In this context, MET has been shown to regulate *igf-1* expression through activation of the GCN2 signaling pathway (Skiba et al., 2014; Hu et al., 2021; Wang et al., 2023). By contrast, the positive effects of TAU appear to be less dependent on such pathways, as its supplementation did not alter tissue MET concentration in totoaba.

Some authors have shown that elevated levels of MET are correlated with increased activity of aspartate aminotransferase (AST), likely due to the conversion of MET to cysteine, followed by its oxidation to cysteine sulfonate and subsequent transformation into pyruvate, which then enters the citric acid cycle (Recasens et al. 1980; Kohlmeier (Kohlmeier, 2003); Yamada et al. 2012). It could be hypothesized that TAU supplementation reduces the dependence on MET, favoring its degradation as an energy source to support intracellular metabolism, a process also documented in mammals (Ito et al. 2015).

The hepatic expression of *csad* observed in this study, along with the final concentration of body taurine in fish fed with D-MET supports the existence of this metabolic pathway. However, similar to other carnivorous species, TAU synthesis in totoaba appears to be limited or inefficient (Goto et al. 2003). Some researches haven’t shown *csad* activity in fish of the Labridae, Scombridae, Soleidae and Rajidae families (Salze and Davis 2015). In carnivorous mammals such as cats, while cysteine sulfinic acid decarboxylase (CSAD) is present, its activity may not suffice to produce adequate amounts of TAU, probably due to competition between the desulfuration and trans-sulfuration pathways (Park and Rogers 1999). An enzymatic assay is needed to confirm whether this limitation also applies to totoaba.

In both mammals and fish, three main pathways have been proposed for TAU synthesis. The cysteic acid pathway, mediated by glutamic acid decarboxylase (GAD), the cysteine sulfinic acid pathway, mediated by (CSAD), and the cysteamine pathway mediated by 2-aminoethanethiol dioxygenase (ADO) (Salze and Davis 2015; Martínez-Burguete et al. 2023) The expression of these pathways varies depending on the organ studied. A tissue-specific analysis could enhance our understanding of TAU´s role as an essential nutrient in totoaba. In this study, the regulation of *csad* by the addition of TAU showed a decrease, which aligns with findings in other marine and freshwater species (Wang et al. 2016a; Ma et al., 2021); Watson 2013). In mammals, *csad* mRNA abundance is influenced by dietary factors such as protein content, availability of sulfur-containing amino acids, and bile salt absorption (Jerkins and Steele, 1992; Jerkins et al. 1998; Kerr et al. 2014). The results suggest that *csad* expression is regulated in a complex manner, with hepatic overexpression linked to MET and TAU deficiency (D-BD), indicating a MET-associated regulatory mechanism independent of TAU concentration. Regarding *taut* expression, it decreased with the supplementation of MET and TAU, suggesting that liver TAU concentration does not exceed a minimum threshold, preventing overexpression of the transporter. Regulation that allows both nutrients to maintain minimum TAU concentrations in hepatocytes. However, further research is needed in specific tissue, as TAU synthesis capacity is tissue-specific (Park 1999; Martínez-Burguete et al. 2023). MET supplementation also resulted in increased total cholesterol in the liver (Table 7), which may significantly relate to its conjugation with TAU and its elimination (Yun et al. 2012).

Viscerosomatic and hepatosomatic indices are commonly linked to excessive lipid accumulation in marine fish, including totoaba (Espe et al. 2010; Zhou et al. 2007; Mata-Sotres et al. 2018; Villasante et al. 2022). However, these indices showed no significant differences across treatments in this study. The latter suggests that adding MET and TAU does not cause hepatic lipid accumulation nor directly affect the observed weight gain differences. Nonetheless, body composition indicates an increase in lipids with TAU supplementation, implying an accumulation of lipid reserves in the muscle. In addition, the hepatosomatic index for totoaba presented higher values than those reported by Fuentes-Quesada et al. (2018), who used soybean meal protein concentrate as a vegetable protein source, instead of conventional soybean meal. In terms of growth parameters, MET supplementation improved the CF and PER, underscoring MET´s importance in protein synthesis, as it is involved in key metabolic pathways related to protein gain (Urbich et al. 2022; Wang et al., 2023). Conversely, no changes in body fat deposition were observed in totoaba fed with supplemented MET, suggesting that the lipogenic effect of this amino acid is not activated at the concentration used in this study (Table 2). This finding contrasts with previous research in other marine species, such as tiger puffer (*Takifugu rubripes*), japanese flounder (*Paralichthys olivaceus*) and cobia (*Rachycentron canadum*), where MET had a direct impact on lipid metabolism and accumulation (Wang et al. 2016a; Xu et al. 2019; Chi et al. 2020).

The lipid content in the liver of fish fed diets supplemented with TAU was similar to levels reported in other studies using high protein content from animal sources (Zapata et al. 2016). Suggesting that the increase in hepatic lipids may be related to the restoration of lipid reserves promoted by the lipogenic and digestive effects generated by TAU supplementation. Moreover, body TAU concentration increased with dietary TAU supplementation, even exceeding levels seen in the BD diet and the MET-supplemented diet. However, MET supplementation did enhance TAU content compared to the BD diet, reinforcing the hypothesis of limited endogenous TAU synthesis capacity in this species. These results are consistent with observations in other carnivorous species, including Japanese flounder, red sea bream and cobia, where dietary TAU deficiency has been linked to specific pathologies, such as steatosis that can led to s green liver condition associated to oxidative stress (Kim et al. 2005; Goto et al. 2001; Watson 2013). Previous studies have reported that TAU supplementation can prevent these conditions by improving lipid storage capacity and promoting a healthier liver acting as antioxidant (Takagi et al., 2005b; Takagi et al. 2006b; Satriyo et al. 2017).

TAU supplementation reduced cholesterol levels in the body, counteracting lipid accumulation. This leads us to hypothesize that body lipid accumulation could dilute cholesterol content. However, the ratio of cholesterol to total fat suggests that the decrease in body cholesterol is due to absolute elimination rather than a dilution effect associated with increased lipid content. The TAU-induced reduction in body and serum cholesterol levels has been documented in turbot *(Scophthalmus maximus* L.) (Yun et al. 2012), where supplementation significantly decreased the concentration of serum cholesterol without changes in liver tissue. At the contrary, in tiger puffer (Xu et al. 2020), an opposite response was observed, showing increases in liver and plasma cholesterol concentrations after TAU supplementation. This discrepancy may be attributed to differences in protein sources used in the diets and variation in the tissues analyzed for cholesterol content. In mammals, TAU has shown to promote cholesterol clearance from the body, both in blood serum (Chen et al. 2012; Tagawa et al. 2022) and in the liver (Park and Lee 1998), establishing it as a key nutrient in regulating cholesterol levels and maintaining liver health. In the present study, the response in the liver was more complex, as MET increased hepatic cholesterol content to 1.2 mg/g (Table 7). Meanwhile, the supplementation of TAU and its combination MET + TAU reduced hepatic cholesterol content, even when normalized to mg/g of lipid, indicating absolute clearance. Notably, this decrease was proportionally smaller than that observed in the whole body, suggesting a possible overexpression of the enzyme HMG-CoA reductase, which regulates cholesterol synthesis and has been previously associated with TAU and MET metabolism in marine fishes (Aguillon et al., 2020; Xu et al. 2020). On the other hand, the increase in hepatic lipid content could be explained by a greater absorption or synthesis of fatty acids and triglycerides, processes in which TAU plays a key functional role (Yun et al. 2012; Iwashita et al. 2009; Yamamoto et al. 2007). The lipogenic effect of TAU, previously reported by Xu et al. (2020), is also supported in this study, confirming its influence on hepatic lipid metabolism.

The regulatory function of MET and TAU likely operates at distinct metabolic levels. TAU contributes to cholesterol elimination via taurocholate synthesis, a pathway described in several marine species (Kortner et al., 2013; Yamamoto et al., 2007). MET, by contrast, affects lipid transport through its involvement in phosphatidylcholine synthesis from S-adenosylmethionine (SAM), which is essential for lipoprotein assembly (Zeisel & Blusztajn 1994; Brosnan & Brosnan 2006; De Geest & Mishra 2023). Moreover, MET is a structural component of apolipoprotein A1 (APOA1), and its oxidation compromises reverse cholesterol transport via ABCA1 (Witkowski et al. 2019; Xu et al. 2022). Taken together, these mechanisms explain why the combined supplementation of TAU and MET resulted in greater cholesterol reduction than either supplement alone (Tab. 6).

In conclusion, combined dietary supplementation of MET and TAU enhanced totoaba growth under low-fishmeal diets. TAU supplementation reduced body cholesterol, while MET tended to raise hepatic cholesterol, although their combination promoted favorable lipid metabolism and higher *igf-1* expression. These findings demonstrate that supplementing sulfur-containing amino acids in balanced proportions not only improves growth performance but also contributes to healthier metabolic regulation in totoaba aquaculture.

## Data Availability

No datasets were generated or analysed during the current study.
